# Special Issue “Design, Synthesis and Applications of Macroporous, Mesoporous, and Microporous Materials”

**DOI:** 10.3390/ijms25137127

**Published:** 2024-06-28

**Authors:** María Teresa Colomer

**Affiliations:** Instituto de Cerámica y Vidrio (ICV), CSIC, c/Kelsen 5, E-28049 Madrid, Spain; tcolomer@icv.csic.es; Tel.: +34-91-735-5840; Fax: +34-91-735-5843

The intention of this Special Issue was to highlight the importance of the design, synthesis, and applications of macro-, meso-, and microporous materials. In this closing editorial, I have described the state of the art of significant macro-, meso-, and microporous materials for both traditional, i.e., well-known, and emerging porous materials. In addition, I would also like to underline the challenges that researchers face nowadays in relation to this topic. In order to achieve these objectives, I have considered several reviews of the recent literature about the design, synthesis and applications of the most relevant types of porous materials.

The classification of these materials is usually given by the diameter of their pores, and according to IUPAC, the diameters for microporous materials are in the range < 2 nm; for mesoporous materials, they range from 2 to 50 nm; and for macroporous materials, the diameters are >50 nm [1].

Porous materials possess a large number of current and potential applications in different fields. They are widely used for gas separation and storage, liquid phase adsorption and catalysis in the fields of food processing, biotechnology, pharmaceuticals, petrochemicals, water remediation, etc. In addition, they are also employed in production and energy storage. For the rational design and synthesis of materials, it is necessary to know the fundamental relationships between the structure, microstructure, and properties. As is well known, the synthesis methods of these membranes influence their physical and chemical features and, in turn, their properties and applications. As Liu and Liu [2] underlined, theoretical scientists can design the structures, predict the properties, and simulate the functionalities of the porous materials by modeling, which can be adjusted with the knowledge of experimental scientists (Figure 1, [2]). Molecular simulations are indispensable for accelerating the development of tailored porous materials. Chemistry provides the knowledge to synthesize new materials and physics provides the knowledge to understand their characteristics and properties for specific applications. Finally, engineers design technological solutions that address industrial needs in different fields. However, a large effort should be made in order to increase the theoretical research that nowadays is still scarce [2]. For instance, inverse design, which is a technique that predicts the compositions and structures of precursor materials based on final functions, can increase the discovery of porous materials. Although progress has been made in the creation of small and simple molecules, in the case of crystalline porous materials, artificial neural networks have not been used so far [3].

Porous materials can be tailored via specific synthesis routes to provide different types of architecture. The optimal membrane microstructure consists of desirable pore size(s), a sharp pore size distribution(s), good pore interconnectivity and porosity, large surface areas and the absence of defects. Liu and Liu [2] indicated that the first structural factor is pore size, which correlates with surface areas and pore volumes and influences the end functions. In this sense, it is worth noting that the synthesis of three-dimensional materials with multiscale pore architecture still represents a current challenge in some aspects. 

As indicated above, porous materials are classified as a function of their pore size, but they can also be classified as a function of their composition in **inorganic, organic, and hybrid porous materials**. In turn, they can be classified as natural or synthetic materials. In addition, in each group, macro-, meso-, microporous, and also hierarchical or ordered hierarchical porous materials can be found. Hierarchical porosity refers to the presence of pores of various size regimes distributed through the whole framework of the porous material. 

## 1. Porous Inorganic Materials

Roy [4] described and analyzed in a recent review the techniques currently used for processing **macroporous ceramics (inorganic materials)** and the influence of the processing parameters on their microstructure and, in turn, on their properties. He also reported the applications of these materials and proposed future research directions in this field. In his review, Roy [4] underlined that the development of a porous component requires an appropriate distribution of properly and adequately sized interconnected pores, considering the nature of the pores (open or closed) and their shape (spherical, elongated, or random). Specific surface area, mechanical strength, corrosion and thermal shock resistance, and other requirements have to be simultaneously considered for a specific application. As is well known, sacrificial template methods can produce porous structures with strict control over the porosity level, pore size and morphology [5,6]. 

**Figure 2:**
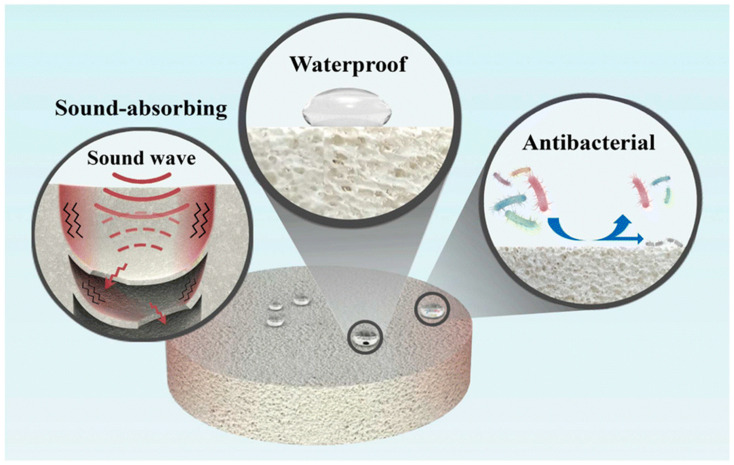
Mechanism of a porous multifunctional ceramic foam. Reprinted with permission from ref. [11]. This article is licensed under a Creative Commons Attribution-NonCommercial 3.0 Unported Licence by Royal Society of Chemistry. Copyright 2024 by Royal Society of Chemistry. Acknowledgement is given to RSC Adv.

Some of the advantages of porous ceramics include low density, high surface area, enhanced thermal insulation, good sound absorption, good permeability, interesting dielectric and piezoelectric properties, etc. Therefore, porous ceramics are very attractive for a wide range of advanced structural and functional applications. For instance, macroporous SiC has been used extensively as volumetric solar receiver [7,8,9,10]; porous ceramics based on Si_3_N_4_ and Al_2_O_3_ are potential candidates as membranes for oil/water separation; and porous zeolite, alumina, silica, etc. are ceramic sound-absorbing materials. Nevertheless, the hydrophilic properties of the latter can cause health risks. According to Zhang et al. [11], the use of ceramic foams with multiple functions can solve this problem. Nowadays, the preparation of this type of material with sound-absorbing, waterproof, and antibacterial properties is being developed (Figure 2, [11]). Porous ceramic materials can also enhance electromagnetic (EM) wave absorption by prolonging the wave propagation path [4]. In addition, the development of solid oxide fuel cells (SOFCs) that can generate high power densities requires improvements in the microstructure of their electrodes. According to Vafaeenezhad et al. [12], the key factors when designing a fuel cell that shows high electrochemical performance are an adequate volume fraction of porosity, pore size and distribution, characteristic pathway diameter, density of triple phase boundaries, and a low tortuosity factor. All of these features aim to maximize the number of electrochemical reaction sites and facilitate gas diffusion [13]. Several sacrificial template methods are capable of generating appropriate porous microstructures and give strict control over the porosity amount, pore sizes and morphology. This type of method includes colloidal crystal templating, polymerization templating (gel-casting), pore-forming agent (pore-former) templating, phase inversion templating and ice templating (freeze-casting) [12,13]. 

**Figure 3:**
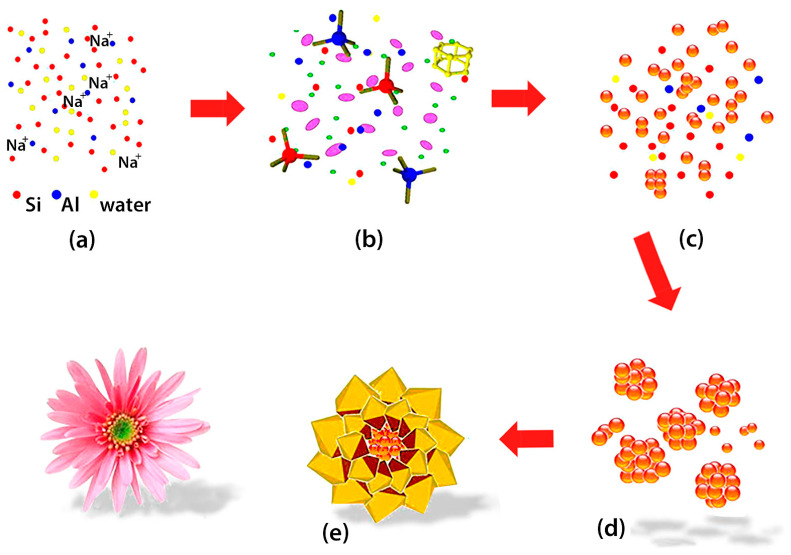
Proposed forming process of the flowerlike Y zeolite. Reprinted with permission from reference [18]. Copyright 2018 by American Chemical Society. Acknowledgment is given to American Chemical Society. According to Du et al. [18], during the initial stage, a high Na^+^ ion concentration in the system contributes to forming smaller zeolite particles by limiting the polymerization extent. Primary nanosized zeolite crystals are therefore formed at that stage (**a**–**c**). At the same time, due to the instability in the hydrothermal synthesis system, the dissociative nanocrystals attract each other and form loose aggregates by self-assembly (**c**,**d**). The inner nanocrystals are difficult to grow because of the confined spaces, while the nanocrystals outer can further grow epitaxially and form the oriented “petals” (**d**,**e**).

**Figure 4:**
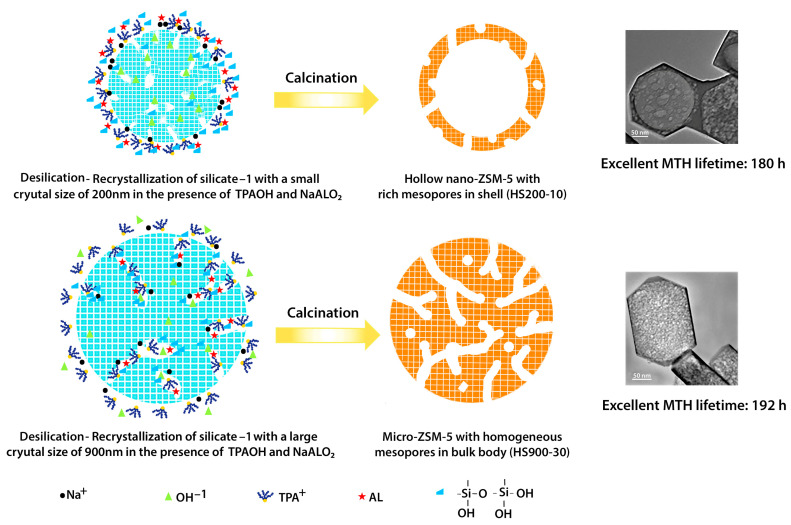
Desilication process (top–down) approach involves post-synthesis modifications to introduce secondary porosity. Reprinted with permission from ref. [19]. Copyright 2019 by American Chemical Society. Acknowledgment is given to American Chemical Society.

In the field of catalysis and in the frame of **porous inorganic materials**, **zeolite minerals** represent a class of microporous aluminosilicates known for their high surface area, chemical stability, adsorption capacity and high ion exchange potential. These crystalline solids possess well-defined structures composed of silicon, oxygen and aluminum, with pores where cations and water are located [14]. They often suffer from diffusion limitations, causing inefficient use of the available catalytically active sites. To solve this problem, different strategies have been used. The first strategy is the development of ordered mesoporous materials, the second one is the synthesis of zeolites with larger pore sizes and the third one is reducing the crystal size to a nanometric range by preparing nanozeolites. Finally, the fabrication of hierarchical zeolites is another strategy. In this latter case, the zeolite presents an additional porous network that can be meso- or/and macroporous on top of and interconnected with the micropores [15]. An increase in the diffusivity is produced thanks to the additional pore networks. It is possible to determine the optimum synthesis conditions to achieve the desired zeolite structure and therefore its properties via a deep understanding of the influence factors on zeolite synthesis. It is not only necessary to improve the efficiency and cost effectiveness of the synthesis but also crucial to establish a solid knowledge for further development [16]. In this way, it will be possible to expand the applications of this type of material. As is well known, zeolites are synthesized using mainly two approaches: bottom-up and top-down methods, each with distinct mechanisms for secondary porous formation. According to Jia et al. [17], the bottom-up approach can imply the use of hard or soft templates, but non-templating processes can also be employed. Hard templating involves the use of rigid templates to guide the formation of zeolite frameworks, while soft templating employs surfactants or organic molecules that self-assemble to create a template structure. In addition, non-templating processes induce secondary porosity via adjustments in synthesis conditions, such as temperature, pH and chemical composition, resulting in the sequential rotational intergrowth of zeolite frameworks. For instance, Du et al. [18] used a hydrothermal process where primary nanocrystals self-assembled into loose aggregates. The inner crystals barely grow due to the reduced space, while the outer crystals grow further to form oriented “petals” (Figure 3, [18]). In contrast, the top-down approach involves post-synthesis modifications to introduce secondary porosity [16]. In this sense, the demetallation technique is a top-down process that removes metal atoms from metal-containing zeolites via chemical treatments like acid leaching or ion exchange (Figure 4, [19]). Such treatments are relatively straightforward and are suitable for use on an industrial scale. According to Ma et al. [19], future prospects in this field imply enhancing the stability and photocatalytic activity of zeolite-based photocatalysts. Doping with non-metal or metal or incorporating additional semiconductors with small band gaps are efficient methods for synthesizing zeolite photocatalysts that absorb visible light. In addition, the combination of zeolite with heterojunction or Z-scheme structures can enhance the separation of photogenerated charge carriers, therefore improving the efficiency of the photocatalyst in environmental care. Challenges remain in the synthesis of zeolites, including impurities in their composition, cost, and production impediments [19]. According to Liaquat et al. [20] and Liang et al. [21], further research for developing homogenous and easily processable hierarchical zeolites for industrial applications, especially in order to prepare more economically and environmentally friendly zeolites, is required.

Traditionally, **mesoporous materials** have been either organic, inorganic, or hybrid materials. Inorganic mesoporous solids such as silicas and metal oxides [22,23] are amorphous and crystalline materials, respectively. Mesoporous silica materials possess high specific surface area (up to 2370 m^2^g^−1^), high specific pore volume (up to 1.4 cm^3^g^−1^), uniform pores, and narrow pore size distributions [22,24]. These characteristics enable mesoporous silicas not only to be excellent supports for active sites in heterogeneous catalysts but also humidity sensors and proton conductors. Mesoporous metal oxides also possess unique structural characteristics such as large pore volume, high surface area and connectivity among pore networks, which make them ideal candidates not only for photocatalysis but also for the latter applications mentioned above for mesoporous silica [23]. In particular, the proton conductivity of micro- and mesoporous silica, metal oxides, and mesoporous glasses has been the subject of growing interest since the 1990s due to their potential as solid electrolytes in humidity sensor devices, fuel cells, etc. [22,23,25,26,27]. It is currently being considered that the presence of an ordered 3-dimensional pore network is advantageous offering a large number of active surface sites and facilitating the accessibility of the reactants and its internal structure to the surrounding media [27]. The enhanced properties can not only be attributed to the increased surface area and uniform pore size distribution but also to the interconnectivity of crystallites in the walls that allows short pathways for charge transport [27]. 

The synthesis methods for creating the above-mentioned membranes and their corresponding films include the sol–gel route, templating approaches (hard/soft templates, bio-molecules foams, etc.) and the self-assembly of crystallites to form 3D morphologies that often look like natural organisms [28,29,30]. The synthesis parameters influence the textural properties of the resulting membranes and films, such as porosity, crystallinity, surface area, etc. Strategies for enhancing the photocatalytic performance in the case of mesoporous titania membranes and films via doping, surface modification and heterostructure formation have been and are currently studied [31,32,33,34,35,36,37,38,39]. These approaches aim to improve the accessibility of the reactants, light absorption, and charge separation in order to optimize the efficiency of the photocatalytic processes using solar energy. Nowadays, the synthesis of bi- and triphasic heterostructures for enhancing both charge separation and charge transfer to achieve higher efficiency in photocatalysis is a hot topic [34]. Up to now, bi- and triphasic mixtures have been synthesized using tedious and expensive synthetic approaches that, in addition, use organic solvents or additives that hinder the large-scale production and commercialization of TiO_2_ and pose an environmental problem [31,32,33,34,35,36,37,38,39]. Thus, simple and greener synthesis routes must be developed and extensively used in order to obtain TiO_2_ with a tailored mixture of phases. Further research and development in this area must lead to the design and fabrication of advanced mesoporous titania materials such as bulk, hollow spheres, films, etc., for a wide range of environmental and energy applications [28,29,39].

As one of the most versatile elements, **carbon materials** present the most abundant number of allotropies composed of pure or mixed hybridization orbitals of sp^1^/sp^2^/sp^3^. They can also be prepared as porous materials. Porous carbon-based materials are a type of inorganic material (PCMs) that are currently a very vast field of research. The design and synthesis of new carbon materials may be stimulated based on a deeper understanding of underlying structures and related properties. The synthetic methods for preparing porous carbon materials with different pore dimensions and pore structures can be classified into two groups, i.e., activation processes and template methods. The activation methods are divided into chemical and physical activation. Chemical modification implies acid or alkali modification, metal modification, and oxidizer modification. In addition, physical activation includes ball milling and gas blowing. The chemical activation method has some advantages, such as low activation temperature, short activation time, high specific surface area, and controllable pore structure. Thus, it is widely used. Porous carbon materials with uniform and interconnected pore sizes, high surface areas and large pore volumes are synthesized by template methods. As is well known, to date, porous carbon materials have been employed as activated carbon (AC), mesoporous carbon, carbon nanotube (CNT), carbon nanofiber, reduced graphene oxide laminate, and nanosheets, among many others. The resulting carbon materials have been used as electrodes for batteries, fuel cells, supercapacitors, and as hosts for the immobilization of biomolecules for biosensors since they possess excellent electrosorption capacity and selectivity for pollutants such as heavy metals, pesticides, and dyes [40,41]. They can also be used as capacitive deionization (CDI) electrodes due to their porosity and good conductance. CDI is currently being widely investigated as an advanced desalination process due to the advantages of eco-friendliness, cost-effectiveness, and high salt removal efficiency. In addition, porous carbon materials derived from metal–organic frameworks (MOFs) show excellent performance in the oxygen reduction reaction (ORR) process [42].

**Figure 5:**
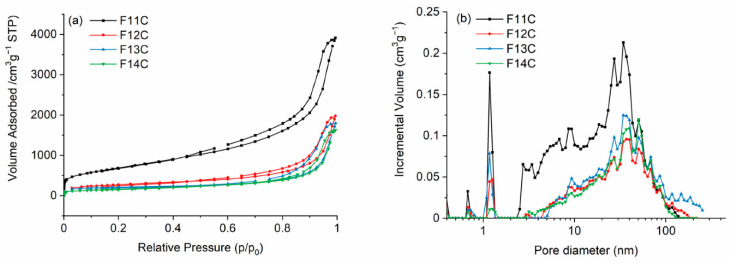
(**a**) N_2_ adsorption–desorption isotherms and (**b**) pore size distribution of SiOC-DC materials pyrolyzed at different temperatures after Cl_2_ etching. FC11 means that the pyrolysis process was performed at 1100 °C, FC12 at 1200, FC13 at 1300, and F14C at 1400 °C, respectively. Reproduced with permission from M.A. Mazo [44]. Published by MDPI (Basel, Switzerland), 2023.

**Figure 6:**
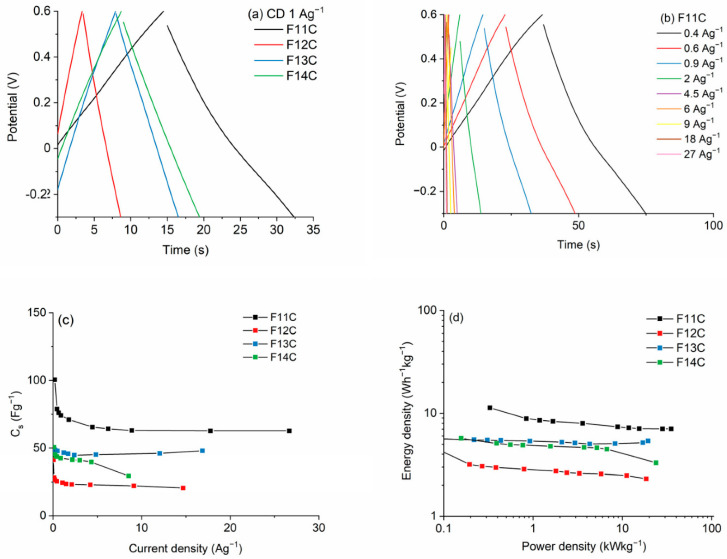
(**a**) GCD curves of SiOC–DC materials pyrolyzed at different temperatures after Cl_2_ etching at a current density of 1 Ag^−1^, (**b**) GCD curves for the F11C sample, (**c**) C_s_ values, and (**d**) Ragone plots, respectively. FC11 means that the pyrolysis process was performed at 1100 °C, FC12 at 1200, FC13 at 1300, and F14C at 1400 °C, respectively. Reproduced with permission from M.A. Mazo [44]. Published by MDPI, 2023.

Carbon-based materials with micro-, meso-, and macropores such as **porous silicon oxycarbide (SiOC), silicon oxycarbide-derived carbon (SiOC-DC), and carbide-derived carbon materials (C-DC)** are in focus due to their potential in energy applications such as gas storage, (H_2_ and CH_4_), CO_2_ capture, electrodes for metal ion batteries (Li, Na, and Zr), and supercapacitors [43]. According to Mazo et al. [43,44], the development of this type of material with micro-, meso-, and macropores enhances the ion accessibility and mass diffusion pathways and hence the specific capacitance *C_s_* and energy density (*E_d_*) values of the related carbon-derived materials (Figure 5 and Figure 6, [44]). The need for more sophisticated hierarchically carbonaceous porous derived materials with the three types of porosity has been carried out with the etching procedure performed using HF or alkaline hydroxides (i.e., NaOH) and has gained importance. HF etching needs lower temperatures and is a softer and more sustainable approach. The use of raw materials for the synthesis of this type of derived carbon materials is not too costly, and both the sol–gel process and HF etching at room temperature could allow for a relatively easy scale up. According to Mazo et al. [44], the samples pyrolyzed at the lowest temperature, i.e., 1100 °C showed planar and less defective carbon domains together with the largest specific surface area and the highest volume of micro–meso–macropores, which upgraded their electrochemical response.

**Nano- and micro-structured scaffolds (NMS)**, such as **single-wall carbon nanotubes (SWCNTs)**, are also used in electrochemical energy storage. According to different authors, thanks to the significant development of nano- and micro-fabrication technology (e.g., templating, 3D printing, etc.) and assembly methods such as magnetic-assisted oriented assembly, it is possible to tailor NMS scaffolds with precise control at the multiscale level [45,46,47,48]. It will allow for the achievement of materials with better properties for their electrochemical applications.

Based on the current development of carbon materials, future research should be focused on the fundamental theory and algorithms for designing the desired properties of carbon materials, including strength, ductility, and optical and electrical properties. 

## 2. Porous Organic and Hybrid Materials 

**Figure 7:**
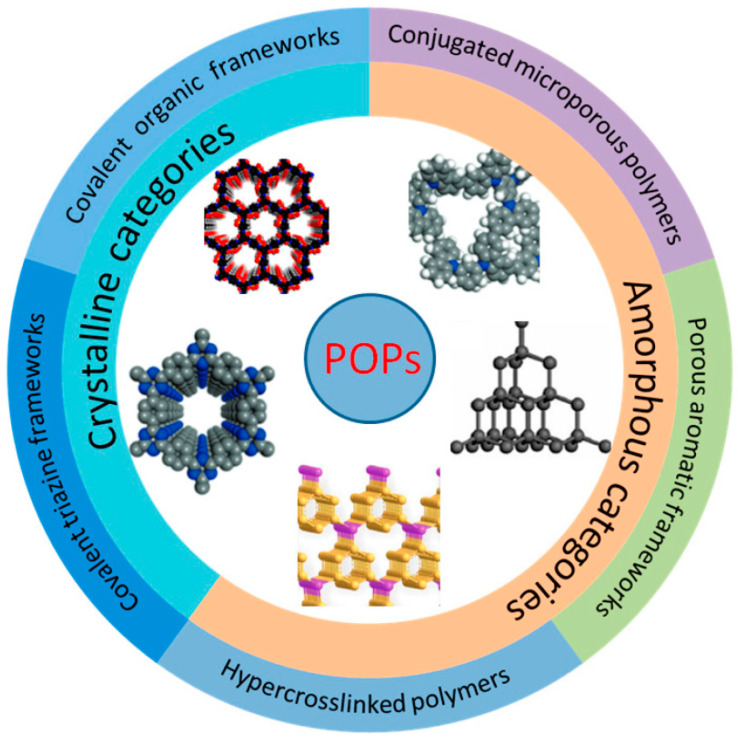
Partial classification of POPs. Reproduced with permission from L. Shao [49]. Published by MDPI, 2023.

**Porous organic polymers (POPs)** can be amorphous or crystalline materials with micro- and mesoporous structures, and their unique physical and chemical properties have made it possible for POPs to achieve good behavior in adsorption, catalysis, sensing, production and energy storage, carbon dioxide (CO_2_) capture, biomedicine, etc. [49]. Figure 7 shows a partial classification of POPs according to Yang et al. [49]. Within the amorphous POPs, conjugated microporous polymers (**CMPs**), porous aromatic frameworks (**PAFs**), and hypercrosslinked polymers (**HPs**) can be found, and within the crystalline class, covalent triazine frameworks (**CTFs**) and covalent organic frameworks (**COFs**) can be found. The functional groups existing on the surface of POPs can provide reaction sites and have the potential to replace metal-based catalysts. At the same time, the stable structure also guarantees the recovery rate of the catalyst and saves costs. According to Yang et al. [49], with existing synthesis technology, achieving stable, uniform and numerous active sites still needs more study. The design of the directional loading of functional groups and active sites, the topological structure and the orderly regulation of the micro-morphology and pore structure are also required in order to improve the catalytic conversion of biomass. In addition, almost all POP materials are synthesized under solvothermal conditions and the requirements for their synthesis environment and equipment make it difficult to achieve large-scale quantities [49]. 

**Mesoporous organic materials** have been prepared by soft templates; in particular, the development of block copolymers has allowed for excellent control of the size and order of mesopores at the molecular level by means of self-assembly and directed assembly [50]. However, control over the micropore sizes of organic polymers remains a challenge. According to Liu et al. [51], for some applications such as gas separation and filtration, it is necessary to prepare a material with different types of pores and, more importantly, to ensure interconnectivity between them. The reduction in tortuosity is still a big challenge nowadays [51]. The applicability of porous organic materials is still limited when they must be used under high or low temperatures, high pressures, and/or high energy radiation [51]. Therefore, the synthesis of novel porous organic materials that can be used under those conditions is an important and wide topic for future research. Finally, and according to Liu and Liu [2], the lack of porous organic materials with uniform pores and homogeneous functional groups hinders experimental checks of the theoretical simulations. 

The field of **porous hybrid materials** is continuously growing since they have a unique combination of inorganic and organic features that generate particular properties. Porous hybrid materials can be obtained by combining POPs such as CMPs, COFs and others with metals, conventional semiconductors, etc. 

**CMPs** are members of porous organic polymers that have distinct π-conjugated skeletons with stable pore structures, ensuring they have large porosity contents. Their synthesis, modification, and potential applications can be found in a recent review by Hayat et al. [52]. According to those authors, these materials demonstrate exceptional charge carrier relocation and mobility over the connection integrity, allowing them to respond rapidly to environmental stimulation or particular chemicals constrained within nanostructures. The research of CMPs in sustainable fuel devices is crucial for the development of CMP-based composites and their use in sustainability and environmental design. Hayat et al. [52] underlined that different methods of developing network routes and synthesized building components provide an enormous heterogeneity of CMPs with various features and design patterns. As a consequence, CMPs have been employed for cathode/anode materials for lithium-ion batteries (LIBs), sodium/potassium LIBs, supercapacitors, fuel cell electrodes for solar cells, hydrogen storage materials, photocatalytic hydrogen and oxygen production reactions (HER and OER processes, respectively), photocatalytic CO_2_ reduction, and so on. Many synthetic methods have been developed in order to obtain this type of material, with the most common routes being solvothermal, ionothermal, mechanochemical, electropolymerization, and microwave methods, and different types of reactions such as Heek, Yamamoto, Schiff-base reactions, etc. [52] In order to improve the efficiency of this type of material, control of the porosity, hydrophilicity, and functionality are required.

**COFs** are crystalline conjugated organic polymers with highly ordered structures, large specific surface areas, stable chemical properties and tunable pore microenvironments. They find applications in various fields such as catalysis, electrochemical and electrochemiluminescent sensing, separation, and energy storage. However, the synthesis of highly conductive COFs is still a challenge. Furthermore, the theoretical prediction of the structure and their electrochemical behavior is not easy. In addition, the potential biotoxicity of COFs must be taken into account before commercialization [53]. 

**MOFs** are crystalline porous hybrid materials built from metal ions/polynuclear building units connected by polytopic organic linkers. They are a novel kind of micro- and mesoporous materials with great specific surface areas and extremely high porosity. MOFs have a unique structure and dispersed active centers therefore, they are good materials as solid catalysts. Several researchers have conducted extensive studies regarding the application of MOF materials for air pollution control. In addition, they have been frequently used in recent years as sacrificial templates for the preparation of carbons, metal oxides, metal nitrides, metal sulfides, and metal phosphides [54,55,56,57,58,59,60,61,62,63,64,65,66,67,68,69]. Common methods for synthesizing MOFs are interfacial diffusion [70], microwave synthesis [71], ultrasonic synthesis [72] and mechanochemical methods [73]. In addition to metal ions and ligands, factors such as solvent, solution concentration, temperature and reaction time, as is expected, play an important role in the crystal structure of MOFs. One of the hot current research fields is monoatomic catalysis using MOFs as precursors. It remains an important line for atmospheric catalysis. The prediction of the performance of MOF-based catalysts via theoretical and computational methodologies with a solid basic theory and optimized standard experimental systems should also be developed. The scale up for future applications should also be taken into account since the current production volume of MOF materials is limited to the laboratory scale. In conclusion, the synthesis of MOF catalysts with cheaper, more stable and more efficient methods should be the focus of future research in this field [70,71,72,73]. 

**Mxenes** is a novel family of 2D transition metal carbides, nitrides, and carbonitrides. They are currently a hot topic due to their amazing physical and chemical properties. These materials can form composites with many compounds, including polymers or metal oxides. It allows us to design MXenes with certain features for a specific application [70]. For instance, **MXene-based composites** have exhibited good performance in desalination and energy storage fields because of their exceptional layered structure, excellent conductivity, high surface area, chemical stability, and good mechanical properties [74,75]. 

Recently, **MXenes** have also been utilized for water splitting and water treatment, i.e., CDI and membranes [76]. By combining MOFs with appropriate materials such as graphene derivatives and Mxenes, composite novel materials can be prepared with improved catalytic performance, thermal and chemical stability, and adsorption capacity. Nevertheless, MXenes are still prepared at the laboratory scale. In addition, there is a need for eco-friendly and cost-efficient synthesis routes for larger-scale applications. Furthermore, the toxic effect on the environment if they are directly released must also be taken into account [76]. 

**Figure 8:**
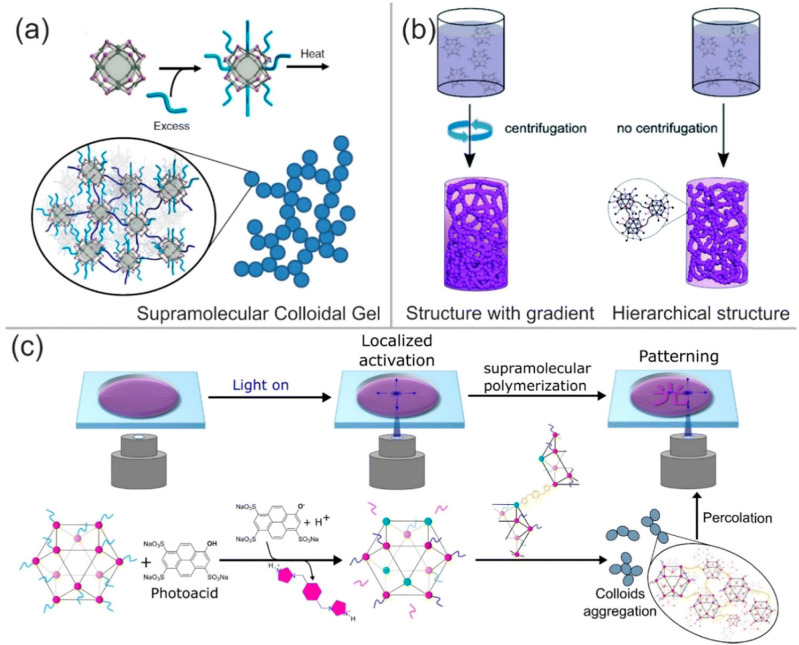
Overview of different design strategies for the formation of MOGs based on crosslinked metal–organic polyhedra. Reprinted with permission from ref. [81]. Copyright 2022 by Royal Soc. Chem. Acknowledgement is given to RSC Adv. In turn, the graph contains figures of the references [78,79,80]. (**a**) Formation of a supramolecular colloidal gel by attachment of excess ditopic linker to the MOP and its removal, which initiates crosslinking of the MOPs. Reprinted without any modification from ref. [78] with permission. Canonical URL. This article is licensed under https://creativecommons.org/licenses/by/4.0/ (accessed on 30 May 2024) Copyright 2018, Springer Nature. Acknowledgement is given to Springer. (**b**) Preparation of a crosslinked MOP-based gel with a gradient. Reproduced with permission without any modification from reference [79]. This article is licensed under a Creative Commons Attribution-NonCommercial 3.0 Unported Licence. Copyright 2019 by Royal Society of Chemistry. Acknowledgement is given to RSC Adv. (**c**) Picture of the formation of a crosslinked MOP gel by localized conversion through deprotonation of a photoacid. Reprinted with permission without any modification from ref [80]. This article is licensed under a Creative Commons Attribution-NonCommercial 3.0 Unported Licence. Copyright 2021 by American Chemical Society. Acknowledgement is given to the American Chemical Society.

**Hierarchical porous metal–organic gels (MOGs) and derived materials** constitute a class of coordination-bond-based soft materials with unique characteristics. The scaffolding framework is fabricated by metal–ligand coordination in combination with other supramolecular interactions. In Figure 8, an overview of different strategies for the design and formation of MOGs is shown [77,78,79,80]. According to Wychowaniec et al. [81], the combination of organic and inorganic (metal/metal–oxo clusters) building blocks will allow us to make significant progress in the development of new electrochemical sensors, superhydrophobic materials, and ion storage devices, among others. Their synthesis, fabrication, and nano-compositing or hybrid formation with other materials have evolved rapidly in the last decade. According to Wychowaniec et al. [81], a crucial point in MOFs and subsequently for MOGs preparation is the precise choice of precursors and reactants, their concentrations, as well as environmental reaction conditions. In addition, one pending subject in the field of MOGs is the exact control of spatial arrangement and morphology, including both the bulk shape/surface area as well as the internal porosity structure. 

**MOGs** possess accessible pores, and thus, they could be used in wastewater treatment with simultaneous molecular sensing or become the next generation of materials for controlled release in medical devices. To achieve these objectives, appropriate processing and reproducibility should be achieved. Thus, more scientific research is required in order to establish the design rules that allow control over the surface chemistry and morphology of this type of material. Wychowaniec et al. [81] predicted that the synthesis of metal oxide-based gels or the preparation of hybrid MOGs using different functional materials will form future strategies for controlling the morphology of new gels. If it is combined with innovative patterning and additive manufacturing techniques, it will allow the formation of gradients of pores in a reproducible and fast way. In addition, and according to Wychowaniec et al. [81], more research on thin films of MOGs compared to other classes of shaped MOGs is necessary. The synthesis of such thin films might be an important issue for the future since it is expected that they could be employed in organic electronics, advanced sensing and biomedical and catalysis applications. 

**Ordered mesoporous organic–inorganic hybrid materials** such as periodic mesoporous organosilicas combine both the respective beneficial properties of the organic and the inorganic constituents, and they often show improved properties that can be expected for a simple mixture of each component [82,83]. Then, they can potentially offer promising features for applications that demand control over the inorganic–organic network and interface, which is critical for adsorption, catalysis, and functional devices and technology. New synthesis approaches to make these materials in a controlled manner are an important hot topic for chemists. According to Ramesha and Meynen [84], the objective is to control the structure, the physical properties, and/or specific chemical reactivity and/or interactions. 

**Porous metal phosphonates** are an important part of the organic–inorganic coordination networks that promise a tremendous potential for material chemists in synthesizing new porous architectures with multifunctional characteristics that can be applied in different fields. According to Ramesha and Meynen [84], heterogeneous catalysis by hybrid metal phosphonates could benefit from the synergy of combining metal centers with tunable organo-functionalities to optimize the interface for catalyzing reactions. The presence of hydrophilic/acidic porous architecture makes metal phosphonates good candidates as proton conductors, solid-acid catalysts, and metal sorption. In the case of separation/extraction applications, the porous amorphous coordination networks could have potential as solid-phase adsorbents, mainly in the fields of separation of lanthanides and actinides from highly acidic solutions, wherein the stability of the material under high acidic conditions and radiolytic stability are key issues. They are also good candidates for solid-phase extraction (SPE). Ramesha and Meynen [84] considered that preparing this type of material as thin films would be useful in order to fabricate fuel cells, sensors, or other devices where ordered channels with specific functional groups are necessary.

Conclusively, and according to the pertinent reviews cited in this text, the progress toward a significant part of porous materials with improved properties requires the synthesis of ordered hierarchical architectures with controlled connectivity between pores with different sizes for current and potential applications. In turn, the development of novel, cheap, and recyclable templates and motifs for self- and directed assembly is also necessary. Although nanosized porous materials promise a very interesting future for numerous applications, important challenges remain. On one hand, the separation process of nanomaterials is not easy and is rather time-consuming, and on the other hand, the yield of the final products is still low. At present, high yield, template-free production, high synthesis efficiency, and green synthesis methods are the key issues of scaling up this type of material. All of this needs collaboration between chemists, material scientists, chemical engineers, physicists, and experts in molecular simulation in order to not only improve the current synthesis methods but also to develop new, greener, and cheaper synthetic routes. The combination of theoretical simulations and experimental observations is crucial for the development of the above-mentioned challenges. It is worth noting that theoretical simulations on this topic are still scarce, and large effort should be made in order to increase this knowledge.

## Figures and Tables

**Figure 1:**
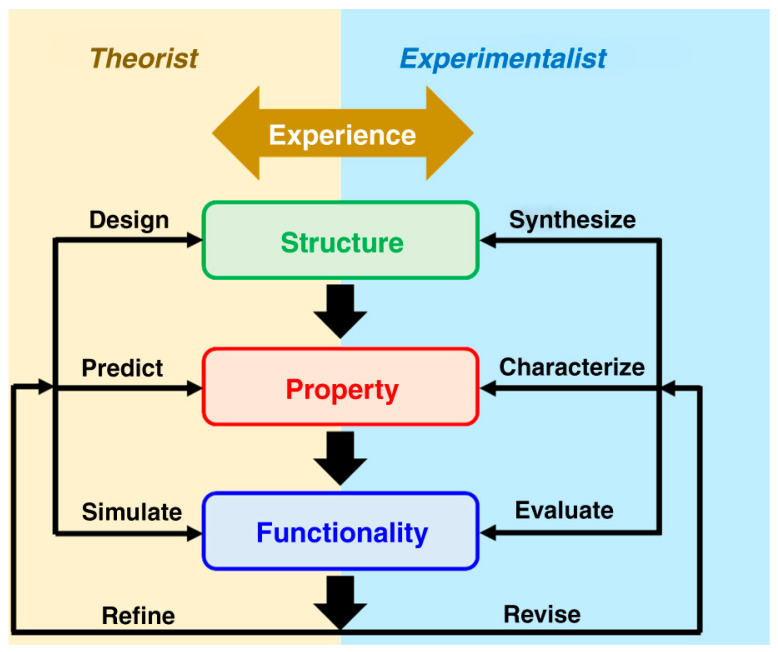
Potential collaboration between theoretical and experimental scientists. Reprinted with permission from ref. [2]. https://creativecommons.org/licenses/by/4.0/ by Nature Publishing Group. URL (accessed on 30 May 2024). Copyright 2020 by the Nature Publishing Group. Acknowledgment is given to the Nature Publishing Group (Berlin, Germany).
